# Supplementary material for: Identification of Crucial Amino Acids in Begomovirus C4 Proteins Involved in the Modulation of the Severity of Leaf Curling Symptoms
Source: Viruses. 2022 Feb 28;14(3):499. doi: 10.3390/v14030499 (PMC8955491; doi:10.3390/v14030499)
Supplement: Supplementary file 1 [file viruses-14-00499-s001.zip › viruses-1586662-supplementary.pdf]

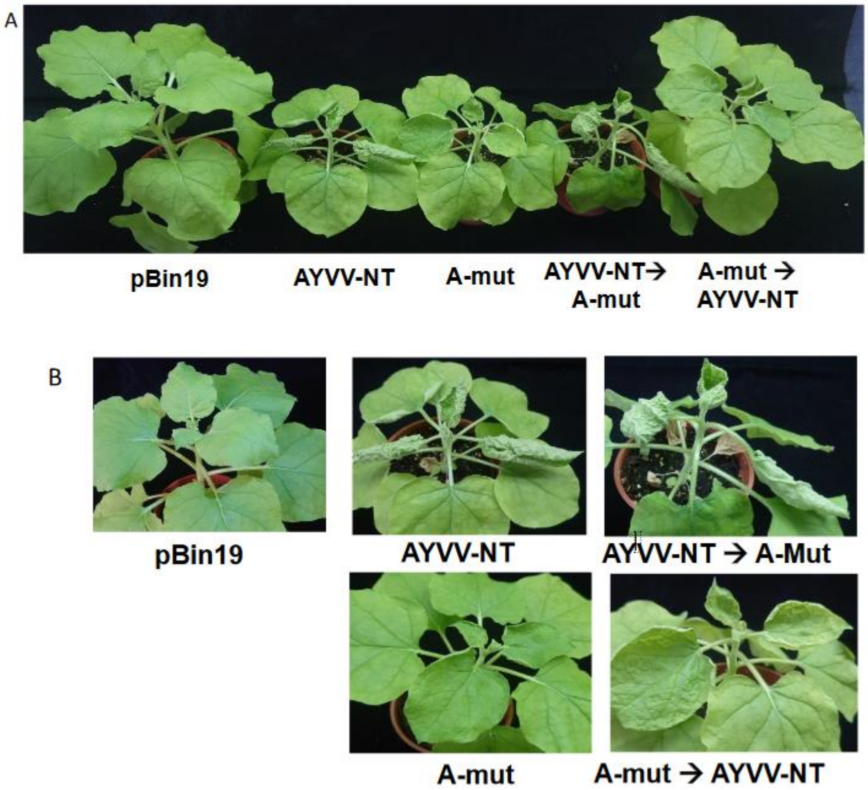

Figure S1. Inoculation assay for testing the potential application of A-mut in the modulation of leaf curling symptoms. To test whether A-mut could be used as a potential "preventive" or "therapeutic" agent for begomovirus induced leaf curling symptoms, *N. benthamiana* plants were first inoculated with AYVV-NT or A-mut, followed by the infiltration of either A-mut or AYVV-NT, respectively, as indicated at the bottom, at 7 dpi, with the arrows representing the order of the infiltration. The effects on leaf curling symptoms were recorded at 34 dpi. (**A**) Overview of the representative phenotypes of plants with different treatments for side-by-side comparison. (**B**) Close-up view of the phenotypes shown in panel A.

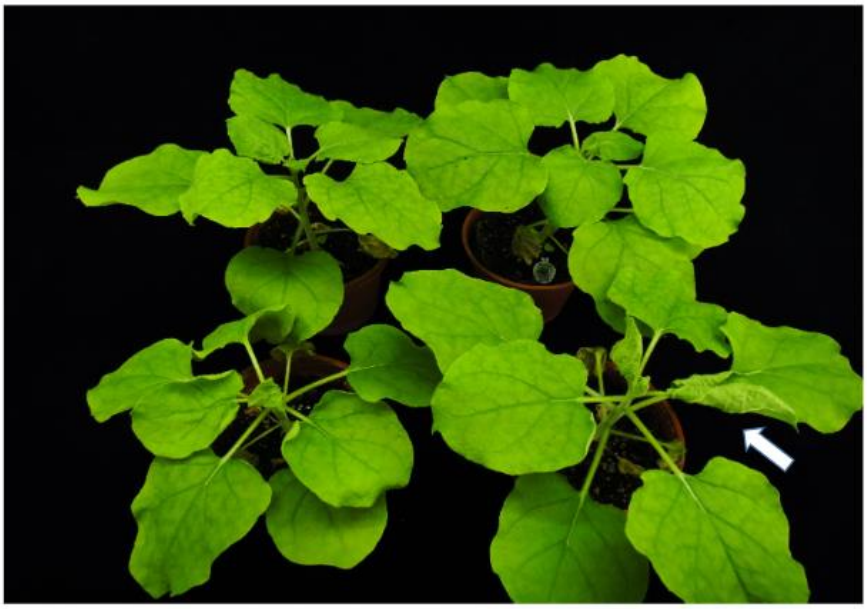

Figure S2. Inoculation assay using A-mut as the preventive agent against the challenge of pBinAYVV. Four *N. benthamiana* plants were first inoculated with A-mut, followed by the infiltration of AYVV-NT, respectively, as indicated at the bottom, at 7 dpi. The effects on leaf curling symptoms were recorded at 34 dpi. The plant at the lower right exhibited severe upward leaf curling symptom following the challenge of pBin-AYVV, as indicated by the white arrow. Sequencing of the progeny viruses from this plant revealed that only AYVV-NT was present. This observation provided evidence suggesting that the "preventive effect" of A-mut was dependent on the successful establishment of A-mut prior to the challenge of pBinAYVV, and not from the wound-induced resistance through the inoculation process.

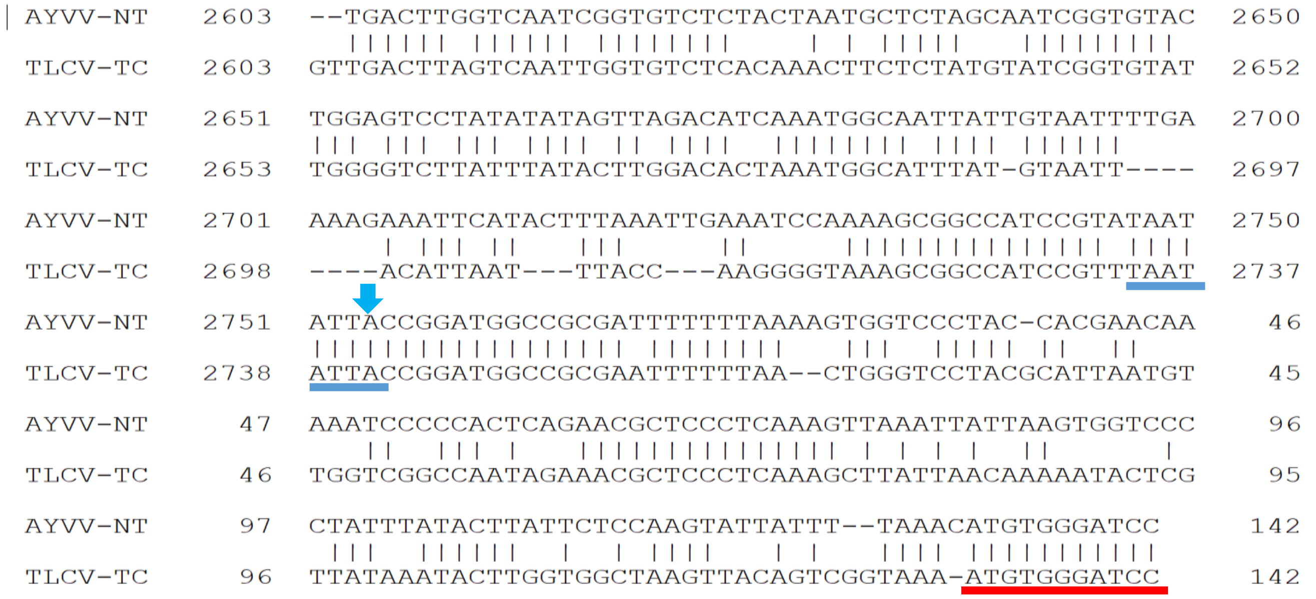

Figure S3. Comparison of nucleotide sequences of the Intergenic Regions (IRs) of AYVV-NT and TLCV-TC. The alignment of IRs (nts 2603 – 131) of AYVV-NT and TLCV-TC is shown. The 5'-terminal sequences (nts 132-142, red-underlined), including the *Bam*HI restriction site, of MP open reading frame are also shown. The conserved nonamer, "TAATATTAC", in the origin of replication (Ori) is indicated by the blue underline. Position 1 of the circular genomes of AYVV-NT and TLCV-TC is indicated by the red arrow.

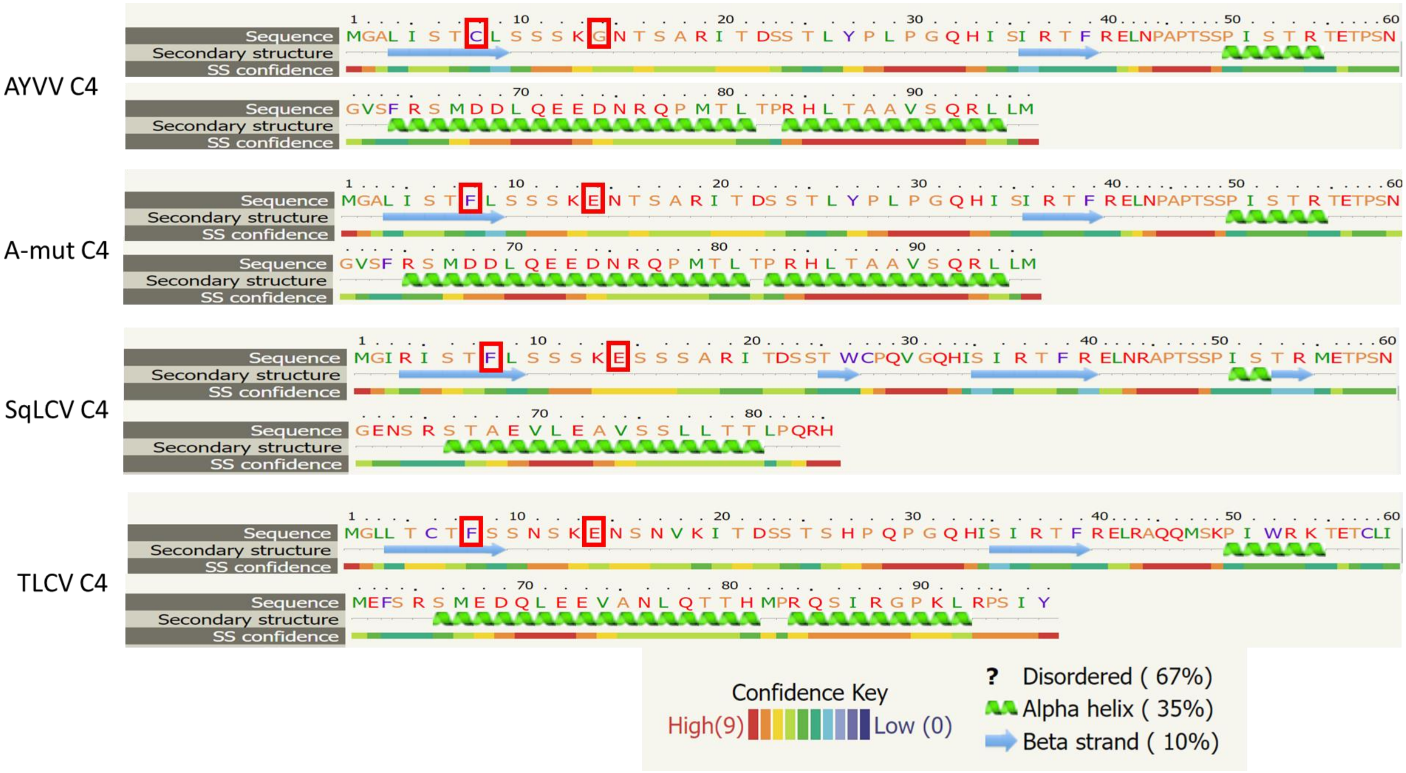

Figure S4. Comparison of the predicted secondary structures of the C4 proteins of AYVV-NT, A-mut, TLCV-TC, and SqLCV-YL. The secondary structures were predicted using the Phyre2 web service (Kelley et al., 2015). The identities of the C4 proteins were indicated on the left of each sequence. The confidence levels and secondary structures were as indicated by the color keys and symbols at the bottom. The amino acids at positions 8 and 14 are highlighted by the red boxes.
